# Burden of treatment-resistant depression in Medicare: A retrospective claims database analysis

**DOI:** 10.1371/journal.pone.0223255

**Published:** 2019-10-10

**Authors:** Dominic Pilon, Kruti Joshi, John J. Sheehan, Miriam L. Zichlin, Peter Zuckerman, Patrick Lefebvre, Paul E. Greenberg

**Affiliations:** 1 Analysis Group, Inc., Montréal, QC, Canada; 2 Janssen Scientific Affairs, LLC., Titusville, NJ, United States of America; 3 Analysis Group, Inc., Boston, MA, United States of America; Chiba Daigaku, JAPAN

## Abstract

**Background:**

Previous studies have assessed the incremental economic burden of treatment-resistant depression (TRD) versus non-treatment-resistant major depressive disorder (i.e., non-TRD MDD) in commercially-insured and Medicaid-insured patients, but none have focused on Medicare-insured patients.

**Objective:**

To assess healthcare resource utilization (HRU) and costs of patients with TRD versus non-TRD MDD or without major depressive disorder (MDD; i.e., non-MDD) in a Medicare-insured population.

**Methods:**

Adult patients were retrospectively identified from the Chronic Condition Warehouse de-identified 100% Medicare database (01/2010-12/2016). MDD was defined as ≥1 MDD diagnosis and ≥1 claim for an antidepressant. Patients initiated on a third antidepressant following two antidepressant treatment regimens of adequate dose and duration were considered to have TRD. The index date was defined as the date of the first antidepressant claim for the TRD and non-TRD MDD cohorts, and as a randomly imputed date for the non-MDD cohort. Patients with TRD were matched 1:1 to non-TRD MDD patients and randomly selected non-MDD patients based on propensity scores. Analyses were also performed for a subset of patients aged ≥65.

**Results:**

Of 29,543 patients with MDD, 3,225 (10.9%) met the study definition of TRD; 157,611 were included in the non-MDD cohort. Matched patients with TRD and non-TRD MDD were, on average, 58.9 and 59.0 years old, respectively. The TRD cohort had higher per-patient-per-year (PPPY) HRU than the non-TRD MDD (e.g., inpatient visits: incidence rate ratio [IRR] = 1.36) and non-MDD cohorts (e.g., inpatient visits: IRR = 1.84, all *P*<0.001). The TRD cohort had significantly higher total PPPY healthcare costs than the non-TRD MDD cohort ($25,517 vs. $20,425, adjusted cost difference = $3,385) and non-MDD cohort ($25,517 vs. $14,542, adjusted cost difference = $4,015, all *P*<0.001). Similar results were found for the subset of patients ≥65.

**Conclusion:**

Among Medicare-insured patients, those with TRD had higher HRU and costs compared to those with non-TRD MDD and non-MDD.

## Introduction

In 2017, the prevalence of major depressive disorder (MDD), a disabling chronic mental health illness, was estimated at 7.1% among adults in the US, corresponding to approximately 17.3 million adults affected by this condition [1]. With approximately 58.4 million beneficiaries in that year, Medicare covered over four million patients with MDD [2]. In 2010, the direct and indirect medical costs associated with MDD exceeded $210 billion in the US [3], and this figure is expected to surpass $280 billion in 2020 [4].

The American Psychiatric Association (APA) guidelines recommend that pharmacotherapy with an antidepressant medication be initiated in patients diagnosed with MDD [5]. However, 25%-50% of patients fail to respond to a first trial of antidepressant [5, 6], and the large US STAR*D study demonstrated that remission rates decrease with each failure of a line of antidepressant therapy [7]. These high rates of antidepressant treatment failure can be the result of a lack of tolerability to medication side effects (e.g., mania and suicidality) or other causes [8, 9]. Treatment-resistant depression (TRD) is most commonly defined as MDD that failed to respond to a minimum of two prior treatments of adequate dose and duration [10], a definition supported by current data [7], although an operational definition endorsed by clinical guidelines is still lacking [10, 11]. Among claims-based studies that used this definition, incidence estimates of TRD among MDD patients varied between 10% and 26% [12–18], with the highest estimates (26%) reported in Medicaid beneficiaries [17, 18]. Recently, it was estimated that the prevalence of TRD may be as high as 31.1% among pharmacologically-treated patients with MDD covered by five different types of insurers [19].

Several studies have evaluated the economic burden of TRD in the US via a systematic literature review [6] and via analyses of health insurance claims databases for various payer channels, including commercially-insured patients [12, 16, 20], Medicaid beneficiaries [17, 18], and the US Veterans Health Administration (VHA) population [21]. These studies invariably found higher costs and healthcare resource utilization (HRU) among patients with TRD relative to those with treatment-responsive MDD (hereinafter referred to as non-TRD MDD) and patients without MDD (hereinafter referred to as non-MDD). However, there is limited data on Medicare-insured patients who, per program eligibility criteria (i.e., individuals ≥65 years of age, with disabilities, or end-stage renal disease [ESRD] [22]), tend to be older than patients benefiting from other types of healthcare insurance.

Although the prevalence and incidence of MDD is lower among older versus younger adults [23–25], elderly patients with depression have a poorer prognosis than younger patients. Indeed, while older adults represent 13% of the general population, they commit 19% of all suicides [5]. Older patients with depression also have higher rates of medical comorbidities and, on average, a higher number of previous depressive episodes which negatively affect prognosis [26]. Moreover, there may be a higher risk of relapse and shorter time to recurrence among older patients with depression relative to younger patients [26]. Thus, an assessment of the burden of illness of TRD in Medicare-insured patients is of interest to inform evidence-based decision making among healthcare payers, policy makers, and providers in an effort to deliver appropriate care and improve care pathways to this growing population. Therefore, the present study sought to assess treatment patterns, HRU, and costs of patients with TRD versus patients with non-TRD MDD or non-MDD patients using the full Medicare-insured population.

## Methods

### Data source

The Chronic Conditions Warehouse de-identified 100% Medicare database (01/2010-12/2016), which contains historical information on patient demographics, plan enrollment, inpatient, outpatient, skilled nursing facility, home health agency, hospice, durable medical equipment, and pharmacy claims was analyzed. The database contains only de-identified information and is thus fully compliant with the requirements of the Health Insurance Portability and Affordability Act (HIPAA).

### Study design

A retrospective longitudinal matched cohort design was used. The index date was defined as the first antidepressant pharmacy claim on or after 07/01/2010 until 12/31/2016 for patients with TRD and non-TRD MDD, and as a randomly imputed date for patients without MDD. Baseline characteristics were evaluated during the six months pre-index date (baseline period). Outcomes and the presence of TRD were evaluated from the index date up until the earliest among two years post-index date, end of continuous eligibility, or end of data availability (hereinafter referred to as the follow-up period).

### Definition of study cohorts

Patients with TRD were compared with patients in two control cohorts: (1) non-TRD MDD patients, and (2) non-MDD patients. MDD patients were considered to have TRD if they failed two antidepressant treatment courses (including augmentation therapy with anticonvulsant, anxiolytic, antipsychotic, lithium, psychostimulant, and thyroid hormone medications) of adequate dose and duration. A dose was defined as adequate if equal to or greater than the minimum starting dose recommended by the APA guidelines [5]. An adequate duration of antidepressant treatment was defined as ≥6 weeks of continuous treatment without gaps longer than 14 days. Failure of a treatment course was defined as a switch of antidepressant (<180 days after the end of the previous treatment), the addition of an antidepressant, or the initiation of an augmentation therapy. Patients with MDD who did not meet these criteria within two years after the index date were classified in the non-TRD MDD cohort. The non-MDD cohort consisted of randomly selected patients without MDD.

### Inclusion criteria

The following inclusion criteria were applied for the TRD and non-TRD MDD cohorts only: (1) ≥1 MDD diagnosis (i.e., International Classification of Diseases, Ninth Revision, Clinical Modifications [ICD-9-CM]: 296.2x [MDD-single episode] and 296.3x [MDD—recurrent episode]; ICD-10-CM: F32.x [excluding F32.8], F33.x [excluding F33.8]), (2) ≥1 claim for an antidepressant between 07/01/2010 and 12/31/2016; (3) ≥1 diagnosis for depression (ICD-9-CM: 296.2x, 296.3x, 300.4x, 311.x, 309.0x, or 309.1x; ICD-10-CM: F32.x, F33.x, F34.1, or F43.21) during the baseline or observation period; (4) no antidepressant claims during the baseline period; and (5) ≥1 antidepressant claim of adequate dose and duration on or after the index date (includes the agent used to define the index date) [5].

The following criteria were applied to all cohorts: (1) no diagnosis for specific psychiatric comorbidities (i.e., psychosis, schizophrenia, bipolar disorder/manic depression, dementia) between 01/01/2010 and 12/31/2016; (2) ≥6 months of continuous eligibility (Medicare Parts A, B, and D coverage without Medicare Part C) prior to the index date (baseline period); (3) ≥6 months of continuous eligibility after the index date; and (4) ≥18 years of age at the beginning of the baseline period.

### Study measures

Baseline characteristics included demographics, physical and mental comorbidities, HRU, and costs. Treatment patterns were evaluated during the follow-up period and included the duration (in days) of antidepressant therapy, the number of different antidepressant agents used, and classes of antidepressants used (i.e., selective serotonin reuptake inhibitors [SSRIs], norepinephrine-dopamine reuptake inhibitors [NDRIs], serotonin-norepinephrine reuptake inhibitors [SNRIs], serotonin modulators, tricyclics and tetracyclics, norepinephrine-serotonin modulators, and monoamine oxidase inhibitors [MAOIs]). All-cause, behavioral health-related, and depression-related HRU and costs were evaluated during the follow-up period. Behavioral health-related HRU and costs were identified using ICD-9-CM codes 290.xx–319.xx, and ICD-10-CM codes F01.xxx-F99.xxx. Depression-related HRU and costs were identified using ICD-9-CM codes 296.2x, 296.3x, 300.4x, 309.0x, 309.1x, 311.xx, and ICD-10-CM codes F32.x, F33.x, F34.1, or F43.21. Primary and secondary diagnosis codes were used. HRU outcomes were stratified into the following categories: inpatient visits, inpatient days, emergency department (ED) visits, outpatient visits, and other visits. Cost outcomes were broken down into pharmacy and medical costs, with the latter category further broken down into inpatient, ED, outpatient, and other costs. Behavioral health-related pharmacy costs included the following classes of agents: anxiolytics, antidepressants, anticonvulsants/mood stabilizers, antipsychotics, and other mood stabilizers (e.g., lithium).

### Statistical analysis

TRD patients were matched 1:1 to non-TRD MDD patients and non-MDD patients using propensity score (PS) models based on key demographics, including age, sex, race, year of the index date, and geographical region. Baseline characteristics were compared across cohorts using standardized differences (std. diff.). Covariates with std. diff. <10% were considered adequately balanced between cohorts. Rates of HRU were compared between matched cohorts using multivariable negative binomial regressions (i.e., incidence rate ratios [IRRs]). Costs were expressed per patient per year (PPPY) in 2017 USD and compared between matched cohorts using multivariable ordinary least squares regressions, with 95% confidence intervals (CIs) and p-values obtained from non-parametric bootstraps with 499 replications. Baseline total all-cause healthcare costs and Quan-Charlson comorbidity index (Quan-CCI) were adjusted for in the multivariable models. Continuous variables were described with means and standard deviations (SDs), and categorical variables were described with frequencies and proportions.

As part of a sensitivity analysis, all aforementioned analyses were performed separately for patients aged ≥65 at the index date in order to separately assess the burden of TRD among a subset of patients who qualified for Medicare based on their age only (as opposed to age, disability status, or ESRD for the main analysis).

## Results

In total, 503,017 patients had ≥1 MDD diagnosis, among which 29,540 were pharmacologically-treated patients with MDD who qualified for inclusion. Of these, 3,224 (10.9%) met the study definition of TRD. Patients with non-TRD MDD (N = 26,316) or non-MDD (N = 157,590) were all matched 1:1 to patients with TRD. In the sensitivity analysis, 1,338 out of 18,908 (7.1%) patients aged ≥65 met the study criteria for TRD.

### Baseline demographic and clinical characteristics

Patient baseline characteristics before matching are presented in **S1 Table** for the main analysis. Matched-on baseline characteristics were adequately balanced between the TRD and non-TRD cohorts after PS matching. Patients in the TRD and non-TRD MDD cohorts were aged, on average, 58.9 and 59.0 years, respectively (std. diff. = 0.9%). Female patients represented 64.0% and 63.6% of the matched TRD and non-TRD MDD cohorts, respectively (std. diff. = 0.8%; **Table 1**). Baseline HRU was higher in the matched TRD cohort than the non-TRD MDD cohort (e.g., number of outpatient visits [mean±SD]: TRD = 9.0±7.1, non-TRD MDD = 7.9±6.7; std. diff. = 15.7%). The mean duration of the observation period was 21.6, 21.6, and 20.4 months in the TRD, non-TRD MDD, and the non-MDD cohort, respectively.

**Table 1:** Baseline characteristics of matched^a^ cohorts (main analysis).

	TRD cohort	Non-TRD MDD cohort	Std. diff.^b^ (%)	Non-MDD control cohort	Std. diff.^b^ (%)
	N = 3,224	N = 3,224		N = 3,224	
**Age at index date (years), mean ± SD [median]**	58.9 ± 14.6 [60]	59.0 ± 14.6 [61]	0.9	59.0 ± 14.6 [61]	0.9
**Female, n (%)**	2,064 (64.0)	2,052 (63.6)	0.8	2,066 (64.1)	0.1
**Race, n (%)**					
White	2,645 (82.0)	2,671 (82.8)	2.1	2,654 (82.3)	0.7
Black	328 (10.2)	326 (10.1)	0.2	328 (10.2)	--
Asian	35 (1.1)	25 (0.8)	3.2	28 (0.9)	2.2
Other/Unknown					
**Year of index date, n (%)**^**c**^					
2011	458 (14.2)	457 (14.2)	0.1	457 (14.2)	0.1
2012	681 (21.1)	679 (21.1)	0.2	678 (21.0)	0.2
2013	497 (15.4)	490 (15.2)	0.6	497 (15.4)	--
2014	475 (14.7)	476 (14.8)	0.1	473 (14.7)	0.2
2015	516 (16.0)	522 (16.2)	0.5	521 (16.2)	0.4
2016	470 (14.6)	475 (14.7)	0.4	470 (14.6)	--
2017	127 (3.9)	125 (3.9)	0.3	128 (4.0)	0.2
**Geographical region, n (%) [27]**					
Northeast	543 (16.8)	540 (16.7)	0.2	545 (16.9)	0.2
Midwest	809 (25.1)	809 (25.1)	--	806 (25.0)	0.2
South	1,308 (40.6)	1,317 (40.8)	0.6	1,312 (40.7)	0.3
West	558 (17.3)	555 (17.2)	0.2	555 (17.2)	0.2
Unknown	<11 (<0.3)	<11 (<0.3)	--	<11 (<0.3)	--
**Quan-CCI, mean ± SD [median] [28]**	1.4 ± 1.6 [1]	1.3 ± 1.5 [1]	6.6	1.0 ± 1.3 [1]	28.9
**Top 5 most frequent physical comorbidities, n (%) [29]**^**d**^					
Hypertension	1,955 (60.6)	1,902 (59.0)	3.4	1,559 (48.4)	24.9
Diabetes	924 (28.7)	937 (29.1)	0.9	795 (24.7)	9.1
Chronic pulmonary disease	909 (28.2)	799 (24.8)	7.7	558 (17.3)	26.2
Deficiency anemias	640 (19.9)	575 (17.8)	5.2	404 (12.5)	20.0
Hypothyroidism	564 (17.5)	537 (16.7)	2.2	425 (13.2)	12.0
**Top 5 most frequent mental comorbidities, n (%) [30]**					
Depression^e^	1,808 (56.1)	1,947 (60.4)	8.8	187 (5.8)	129.6
Anxiety disorders	1,016 (31.5)	879 (27.3)	9.3	245 (7.6)	63.2
Sleep-wake disorders	764 (23.7)	658 (20.4)	7.9	336 (10.4)	35.9
Substance-related and addictive disorders	702 (21.8)	613 (19.0)	6.9	313 (9.7)	33.6
Other conditions that may be a focus of clinical attention	500 (15.5)	446 (13.8)	4.7	235 (7.3)	26.1
**Baseline costs and resource use**					
**Had ≥1 healthcare visit/service, n (%)**					
Inpatient	825 (25.6)	705 (21.9)	8.8	324 (10.0)	41.5
ED	1,109 (34.4)	961 (29.8)	9.8	592 (18.4)	37.0
Outpatient	3,030 (94.0)	3,050 (94.6)	2.7	2,848 (88.3)	20.0
Other	1,729 (53.6)	1,578 (48.9)	9.4	1,454 (45.1)	17.1
**Total healthcare costs (US $2017), mean ± SD [median]**	26,498 ± 57,243 [7,236]	22,064 ± 54,182 [5,215]	8.0	11,564 ± 27,935 [3,17]	33.2
Medical costs	23,745 ± 56,246 [5,098]	19,403 ± 52,085 [3,380]	8.0	9,094 ± 25,439 [1,749]	33.6
Pharmacy costs	2,753 ± 8,779 [950]	2,661 ± 9,751 [697]	1.0	2,470 ± 9,568 [543]	3.1

**Abbreviations:** ED = emergency department; MDD = major depressive disorder; Quan-CCI = Quan-Charlson comorbidity index; SD = standard deviation; Std. diff. = standardized difference; TRD = treatment-resistant depression

Notes

^a^Patients were matched on propensity score (the probability of being in the TRD cohort vs. the non-TRD MDD or non-MDD cohort), generated using probability estimates from a logistic regression model adjusted for categorical age, sex, race, year of the index date, geographical region, and type of healthcare plan

^b^For continuous variables, the standardized difference is calculated by dividing the absolute difference in means of the control and the TRD cohorts by the pooled standard deviation of both groups. The pooled standard deviation is the square root of the average of the squared standard deviations. For dichotomous variables, the standardized difference is calculated using the following equation where P is the respective proportion of participants in each group: (PTRD-Pcontrol)/√[(PTRD(1-PTRD)+Pcontrol(1-Pcontrol))/2].

^c^The index date was defined as the date of the first prescription fill for an antidepressant.

^d^The top 5 most frequent Elixhauser comorbidities identified in the TRD cohort were reported.

^e^Depression diagnoses included the following diagnoses ICD-9-CM: 296.2x (MDD—single episode), 296.3x (MDD—recurrent episode), 300.4x (dysthymic disorder), 309.0x (adjustment disorder with depressed mood), 309.1x (prolonged depressive reaction), and 311.x (depressive disorder, not elsewhere classified) or ICD-10-CM: F32x (MDD—single episode), F33x (MDD—recurrent episode), F341 (dysthymic disorder) and F4321 (adjustment disorder with depressed mood).

In the sensitivity analysis performed among patients aged ≥65, mean age was 72.6 years both in the TRD and non-TRD MDD cohorts, and other matched-on covariates were well balanced between cohorts. Quan-CCI (mean±SD: TRD = 1.7±1.7, non-TRD MDD = 1.5±1.6; std. diff. = 11.8%), baseline HRU (e.g., number of outpatient visits [mean±SD]: TRD = 10.4±7.4, non-TRD MDD = 8.8±6.7, std. diff. = 23.4%) and baseline total healthcare costs (mean±SD: TRD = $31,404±$60,120, non-TRD MDD = $23,239±$49,614, std diff. = 14.8%) were higher in the TRD cohort than the non-TRD MDD cohort.

### Treatment patterns

**Fig 1:**
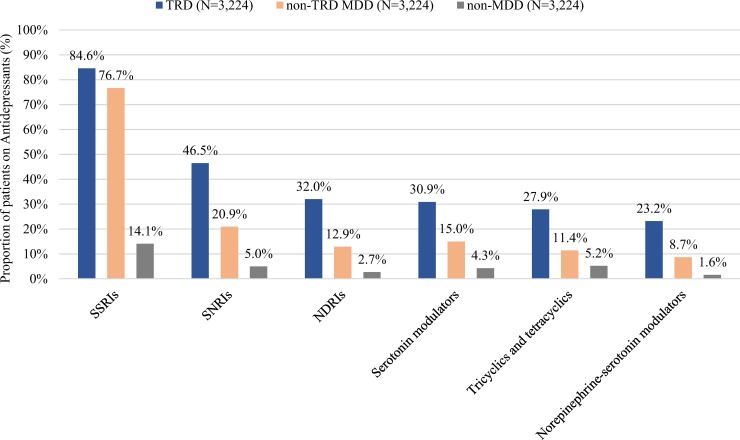
Antidepressant treatment patterns observed during the follow-up period for the TRD, non-TRD MDD, and non-MDD cohorts (main analysis). **Abbreviations:** MDD = major depressive disorder; NDRI = norepinephrine-dopamine reuptake inhibitors; SSRI = selective serotonin reuptake inhibitors; SNRI = serotonin-norepinephrine reuptake inhibitors; TRD = treatment-resistant depression.

For the TRD versus non-TRD MDD cohorts, the most frequently used antidepressants classes were SSRIs (84.6% vs. 76.7%), SNRIs (46.5% vs. 20.9%), NDRIs (32.0% vs 12.9%), tricyclics and tetracyclics (27.9% vs. 11.4%), and norepinephrine-serotonin modulators (23.2% vs. 8.7%; Fig 1). The use of antidepressants was low in non-MDD patients (e.g., SSRIs: 14.1%, tricyclics and tetracyclics: 5.2%, SNRIs: 5.0%; Fig 1). Duration of antidepressant therapy was longer among patients with TRD (265.2 days) relative to those with non-TRD MDD (194.0 days, *P*<0.001). The number of unique antidepressant agents received was higher in the TRD cohort relative to the non-TRD MDD cohort (mean±SD: TRD = 3.0±1.1, non-TRD MDD = 1.6±0.8, *P*<0.001). Similar treatment patterns were observed in the sensitivity analysis among patients aged ≥65 years (S1 Fig).

### Healthcare resource utilization

**Fig 2:**
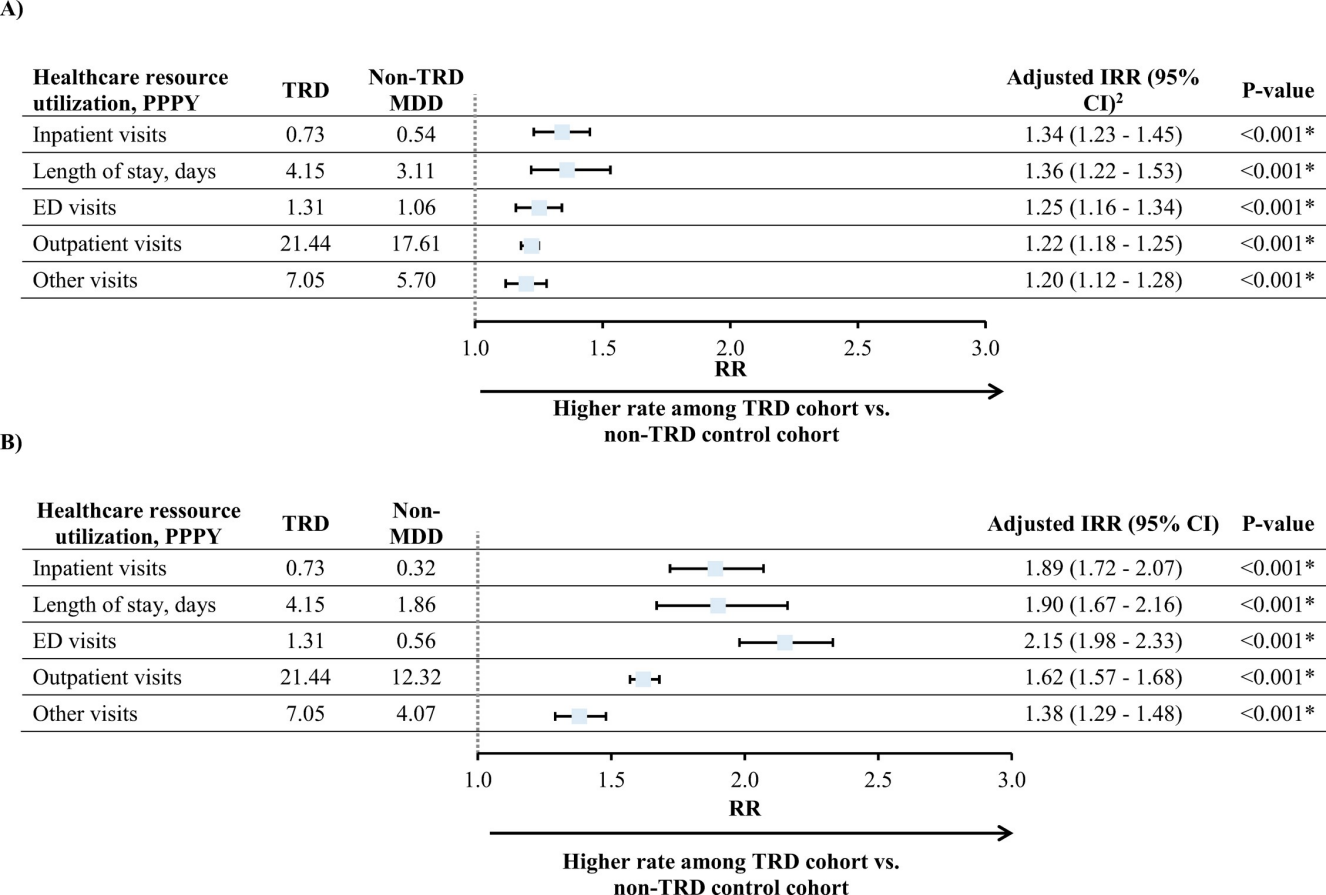
All-cause HRU per-patient-per-year (main analysis)^a^. **A)TRD versus non-TRD MDD. B) TRD versus non-MDD. Abbreviations:** CI = confidence interval; IRR = incidence rate ratio; MDD = major depressive disorder; PPPY = per patient per year. **Notes:** [1] HRU was measured from the index date up to two years post-index date. [2] Adjusted for baseline total healthcare costs and Quan-CCI.

During the observation period, patients with TRD had 34% and 36% higher adjusted rates of all-cause PPPY inpatient visits and days of inpatient stays, respectively, relative to non-TRD MDD patients (all *P*<0.001; Fig 2). When using the non-MDD cohort as comparator, these differences reached 89% and 90%, respectively (all *P*<0.001; Fig 2). Similarly, the rates of all-cause PPPY ED visits were 25% higher in the TRD cohort than the non-TRD MDD (*P*<0.001), and 115% higher when compared to the non-MDD cohort (*P*<0.001; Fig 2). Similar results were obtained for all-cause outpatient and other visits, although the magnitude of the difference was numerically lower (Fig 2).

The TRD cohort had higher rates of PPPY behavioral health-related HRU relative to the non-TRD MDD cohort (e.g., inpatient visits: IRR = 1.49) and versus the non-MDD cohort (e.g., inpatient visits: IRR = 3.84, all *P*<0.001). Depression-related HRU was also significantly higher in the TRD cohort relative to either comparator group (e.g., inpatient visits for TRD vs. non-TRD MDD: IRR = 1.53, *P*<0.001; **S2 Table**). In the sensitivity analysis, similar results were obtained among patients ≥65, although the magnitude of the difference in all-cause PPPY ED visits appeared higher (i.e., IRR = 1.38, *P*<0.001; **S3 Table**).

### Costs

**Fig 3:**
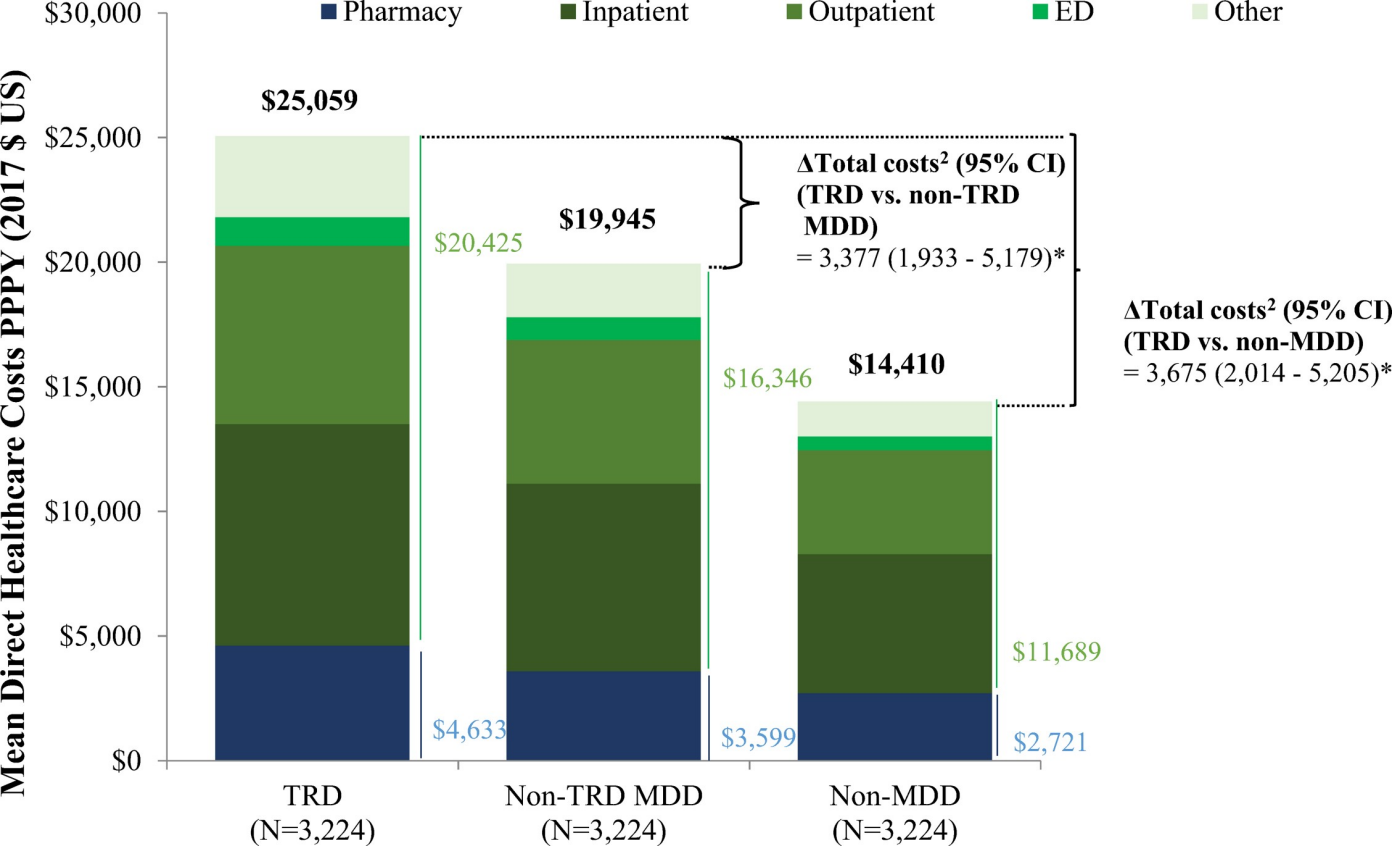
All-cause healthcare costs per-patient-per-year^a^ during the follow-up period (main analysis). **Abbreviations:** CI = confidence interval; ED = emergency department; MDD = major depressive disorder; PPPY = per patient per year; TRD = treatment-resistant depression. **Notes:** *: significant at the 5% level. [1] Healthcare costs measured from the index date up to 2 years post-index date. [2] Adjusted for baseline total healthcare costs and Quan-CCI.

During the observation period, the total all-cause PPPY healthcare costs were $25,059 in the TRD cohort, $19,945 in the non-TRD MDD cohort, and $14,410 in the non-MDD cohort (all *P*<0.001; Fig 3 and Table 2). The TRD cohort had significantly higher adjusted PPPY all-cause healthcare costs versus the non-TRD MDD (adjusted cost difference = $3,377, *P*<0.001) or non-MDD cohorts (adjusted cost difference = $3,675, *P*<0.001; Fig 3 and Table 2).

**Table 2:** All-cause, behavioral health-related, and depression-related healthcare costs per-patient-per-year during the follow-up period (main analysis).

Healthcare cost (US $2017) per patient per year (PPPY)	Mean ± SD [median]			Adjusted cost difference (95% CI); P-value^a^^,^^b^	
	TRD cohort (N = 3,224)	Non-TRD MDD cohort (N = 3,224)	Non-MDD cohort (N = 3,224)	Non-TRD MDD cohort	Non-MDD cohort
**All-cause medical and pharmacy costs**	25,059 ± 36,832 [12,26]	19,945 ± 40,138 [7,795]	14,410 ± 34,446 [3,902]	3377 (1,933 ; 5,179); <0.001*	3675 (2,014 ; 5,205); <0.001*
** All-cause medical costs**	20,425 ± 33,463 [8,561]	16,346 ± 36,808 [5,229]	11,689 ± 31,907 [2,499]	2555 (1,097 ; 4,133); <0.001*	2700 (1,160 ; 4,143); <0.001*
Inpatient costs	8,886 ± 20,930 [0]	7,524 ± 27,935 [0]	5,580 ± 24,575 [0]	481 (-664 ; 1,638); 0.421	-27 (-1,341 ; 1,112); 0.990
ED costs	1,161 ± 2,491 [343]	918 ± 3,144 [144]	548 ± 3,172 [0]	182 (27 ; 348); 0.040*	459 (324 ; 590); <0.001*
Outpatient costs	7,143 ± 11,410 [4,14]	5,766 ± 12,178 [2,809]	4,171 ± 9,862 [1,512]	988 (447 ; 1,541); <0.001*	1283 (802 ; 1,777); <0.001*
Other costs	3,236 ± 10,821 [429]	2,138 ± 5,827 [208]	1,391 ± 5,283 [77]	904 (540 ; 1,331); <0.001*	985 (619 ; 1,359); <0.001*
** All-cause pharmacy costs**	4,633 ± 10,843 [1,985]	3,599 ± 10,472 [1,319]	2,721 ± 10,163 [647]	822 (346 ; 1,372); 0.004*	974 (373 ; 1,517); <0.001*
**Behavioral health-related medical and pharmacy costs**	7,698 ± 14,081 [2,248]	5,437 ± 16,695 [759]	1,991 ± 9,814 [0]	1899 (1,174 ; 2,589); <0.001*	4526 (3,972 ; 5,065); <0.001*
**Behavioral health-related medical costs**^**c**^	7,021 ± 14,025 [1,374]	5,180 ± 16,673 [463]	1,837 ± 9,744 [0]	1482 (751 ; 2,174); <0.001*	4026 (3,470 ; 4,567); <0.001*
Inpatient costs	4,689 ± 11,191 [0]	3,657 ± 14,434 [0]	1,463 ± 9,084 [0]	741 (115 ; 1,339); 0.016*	2304 (1,818 ; 2,770); <0.001*
ED costs	424 ± 1,555 [0]	275 ± 1,668 [0]	106 ± 685 [0]	129 (33 ; 212); 0.012*	283 (230 ; 347); <0.001*
Outpatient costs	948 ± 2,248 [332]	638 ± 2,044 [135]	128 ± 689 [0]	294 (199 ; 407); <0.001*	762 (679 ; 847); <0.001*
Other costs	960 ± 4,205 [0]	611 ± 2,737 [0]	141 ± 1,308 [0]	318 (147 ; 494); <0.001*	677 (544 ; 819); <0.001*
**Psychiatric pharmacy costs**^**d**^	677 ± 1,528 [252]	256 ± 862 [49]	155 ± 1,050 [0]	417 (360 ; 476); <0.001*	500 (434 ; 565); <0.001*
**Depression-related pharmacy and medical costs**	5,106 ± 11,306 [912]	3,690 ± 12,596 [288]	424 ± 2,839 [0]	1153 (597 ; 1,703); <0.001*	4027 (3,661 ; 4,373); <0.001*
**Depression-related medical costs**^**e**^	4,815 ± 11,303 [473]	3,556 ± 12,590 [154]	385 ± 2,817 [0]	999 (449 ; 1,550); <0.001*	3780 (3,407 ; 4,128); <0.001*
Inpatient visits	3,210 ± 9,142 [0]	2,502 ± 11,099 [0]	273 ± 2,435 [0]	494 (-34 ; 968); 0.060	2444 (2,154 ; 2,756); <0.001*
ED visits	237 ± 1,143 [0]	142 ± 1,031 [0]	18 ± 244 [0]	81 (23 ; 139); 0.012*	195 (161 ; 235); <0.001*
Outpatient costs	603 ± 1,716 [156]	414 ± 1,727 [57]	31 ± 385 [0]	177 (100 ; 267); <0.001*	538 (479 ; 602); <0.001*
Other costs	767 ± 3,718 [0]	498 ± 2,352 [0]	63 ± 799 [0]	248 (100 ; 390); <0.001*	603 (480 ; 724); <0.001*
**Antidepressant pharmacy costs**	290 ± 561 [110]	134 ± 325 [27]	39 ± 192 [0]	154 (131 ; 178); <0.001*	247 (226 ; 268); <0.001*

**Abbreviations:** CI = confidence interval; ED = emergency department; SD = standard deviation

Notes

*: significant at the 5% level

^a^ Unadjusted cost differences were estimated using an ordinary least squares regression model and 95% CIs and p-values were estimated using a non-parametric bootstrap procedure (N = 499).

^b^ A cost difference > 0 indicates that the TRD cohort had higher costs than non-TRD MDD cohort.

^c^ Behavioral health-related medical costs were defined as all costs during a visit with any of the following ICD-9 CM diagnostic codes: 290.xx– 319.xx and their ICD-10 CM equivalent.

^d^ Psychiatric pharmacy costs include the following classes of agents: antidepressants, anxiolytics, anticonvulsants/mood stabilizers, antipsychotics, and other mood stabilizers (e.g., lithium).

^e^ Depression-related medical costs were identified using the following ICD-9 CM diagnosis codes: 296.2x, 296.3x, 300.4x, 309.0x, 309.1x, 311.xx and their ICD-10 CM equivalents.

All-cause PPPY medical costs drove the majority of the cost difference whether TRD patients were compared to those with non-TRD MDD or non-MDD (% of total adjusted cost difference: vs. TRD = 75.7%, vs. non-MDD = 73.5%); outpatient costs were the main driver for both comparisons (Table 2). Similar patterns and cost drivers were observed when assessing total behavioral health-related costs (% of total all-cause costs: TRD = 30.7%, non-TRD MDD = 27.3%, non-MDD = 13.8%) and total depression-related costs (% of total all-cause costs: TRD = 20.4%, non-TRD MDD = 18.5%, non-MDD = 2.9%; Table 2).

In the sensitivity analysis, similar results were found among patients ≥65 years of age for the TRD versus non-TRD MDD comparison (adjusted cost difference = $4,524) and TRD versus non-MDD comparison (adjusted cost difference = $7,126, all *P*<0.001; **S4 Table**). Trends similar to those observed in the main analysis were observed with respect to the drivers of this cost difference (**S4 Table**).

## Discussion

In this retrospective, claims-based study, the burden of TRD was assessed in a population of Medicare-insured patients. All categories of HRU assessed were significantly increased in patients with TRD relative to those with non-TRD MDD, with all-cause inpatient and ED visits having the largest difference in magnitude. Patients with TRD incurred higher total all-cause PPPY healthcare costs of $25,059 relative to only $19,945 and $14,410 for patients with non-TRD MDD and non-MDD, respectively, yielding adjusted cost differences of $3,377 and $3,675.

In the present study, 10.9% of pharmacologically-treated patients with MDD met the definition of TRD in the main analysis, and 7.1% did so in the sensitivity analysis performed among patients aged ≥65. These figures are lower than those reported in previous studies which applied the same study cohort definitions to other populations, including commercially-insured patients (16.2%) [12] and Medicaid beneficiaries (26.1%) [18]. While this may indicate that the prevalence of TRD among Medicare-insured patients with MDD is lower than that among other populations, the presence of multiple mental and physical comorbidities can complicate the management of TRD in older patients and underlie this result. Indeed, due to concerns over potential side effects, comorbidities (e.g., hepatic or renal impairment), or drug-drug interactions [5, 31], some patients may not be offered further lines of antidepressant therapy, and would consequently not be identified as having TRD based on the present algorithm. More research is warranted to better understand this apparent lower prevalence of TRD in Medicare-insured patients versus other populations.

With respect to treatment patterns, the Medicare-insured patients with TRD included in the present study received, on average, a lower number of different antidepressants compared with commercially-insured and Medicaid-insured patients included in previous studies (i.e., mean: Medicare = 3.0, commercial = 3.3, Medicaid = 3.3) but for a longer duration (mean [days]: Medicare = 265, commercial = 235, Medicaid = 224) [12, 18]. Average treatment duration was even longer in the sensitivity analysis performed among patients ≥65 years of age (281 days). This supports the possibility that tolerable treatment options are limited in elderly patients due to the high rates of comorbidities in this population [31], which may lead to reduced rates of treatment switching. Additionally, the APA guidelines recommend that older or medically compromised patients receive a lower starting therapeutic dose (i.e., 50% of standard dose) followed by dose escalation [5]. Thus, the longer time of antidepressant therapy in this elderly population may partially reflect the longer time required for the sequential escalation of antidepressant doses. Moreover, the most common class of antidepressants was SSRIs, which is consistent with previous findings in commercially-insured [12, 16] and Medicaid-insured [18] patients.

With respect to costs, the adjusted total healthcare cost difference is in line with the vast body of literature showing that TRD patients incur significantly higher costs compared with non-TRD MDD patients [6, 12, 16–18, 20]. However, the difference appeared smaller than that observed in different populations (Medicare = $3,675, commercial = $6,709, Medicaid = $4,382) [12, 18], suggesting the incremental cost burden of TRD may be lower in Medicare-insured patients.

Total healthcare costs were higher among the Medicare-insured patients with TRD included in the present study relative to those included in previous studies that assessed commercially-insured and Medicaid-insured patients (mean: Medicare = $25,059, commercial = $17,261, Medicaid = $16,654) [12, 18]. This difference was almost entirely driven by higher total medical costs relative to those found in other populations (mean: Medicare = $20,425; commercial = $13,795, Medicaid = $12,403), which may be explained by other co-occurring medical conditions in this older population. In addition, behavioral health-related costs accounted for only a minority (~30%) of total healthcare costs in the TRD cohort, an observation consistent with several previous studies, which reiterates the importance of comorbidities as a major contributor to the economic burden of TRD [12, 16, 18].

## Limitations

The present study is subject to some limitations. First, the algorithm used to identify patients with TRD relied solely on pharmacy claims, and clinical considerations to specifically assess treatment failure, response, and remission could not be incorporated. Second, although adjustment techniques such as matching and multivariable model adjustments were used to minimize potential confounding, comparisons may be subject to unmeasured confounders. Third, analyses are subject to inherent limitations of claims databases such as inaccuracies due to coding errors and missing data. Fourth, included patients may not be representative of the entire TRD or MDD population, given that patients with concurrent diagnoses of psychosis, bipolar, manic disorder, schizophrenia, or dementia were excluded from the study to ensure that the antidepressant was used to treat MDD. In addition, due to the inclusion of patients who received an antidepressant treatment course of adequate dose and duration, non-adherent patients may be underrepresented in the study sample.

## Conclusion

In the present retrospective claims-based study, Medicare-insured patients with TRD were found to have significantly higher HRU and costs compared to patients with non-TRD MDD and those without MDD. These results are in line with the growing body of literature which highlights the significant incremental burden of TRD versus non-TRD MDD across multiple different populations [12, 16–18, 20, 21]. Further research is needed to identify and validate potential modifiable (e.g., cholesterol [32, 33]) and non-modifiable (e.g., depressive illness burden, concurrent psychiatric and general medical disorders [7]) factors associated with TRD in Medicare-insured patients, which may enable the identification of patients who may benefit the most from antidepressant treatment.

## Supporting information

S1 Fig Antidepressant treatment patterns observed during the follow-up period for the TRD, non-TRD MDD, and non-MDD cohorts for the subset of patients aged ≥65. **Abbreviations:** MDD = major depressive disorder; NDRI = norepinephrine-dopamine reuptake inhibitors; SSRI = selective serotonin reuptake inhibitors; SNRI = serotonin-norepinephrine reuptake inhibitors; TRD = treatment-resistant depression.
(TIF)

S1 Table Patient baseline characteristics (main analysis) before matching. **Abbreviations:** ED = emergency department; MDD = major depressive disorder; Quan-CCI = Quan-Charlson comorbidity index; SD = standard deviation; Std. diff. = standardized difference; TRD = treatment-resistant depression. **Notes:** ^a^ For continuous variables, the standardized difference is calculated by dividing the absolute difference in means of the control and the TRD cohorts by the pooled standard deviation of both groups. The pooled standard deviation is the square root of the average of the squared standard deviations. For dichotomous variables, the standardized difference is calculated using the following equation where P is the respective proportion of participants in each group: (PTRD-Pcontrol)/√[(PTRD(1-PTRD)+Pcontrol(1-Pcontrol))/2]. ^b^ The index date was defined as the date of the first prescription fill for an antidepressant. ^c^ Based on U.S. census regions (http://www2.census.gov/geo/pdfs/maps-data/maps/reference/us_regdiv.pdf). ^d^ Quan H, Sundararajan V, Halfon P et al. Coding Algorithms for Defining Comorbidities in ICD-9-CM and ICD-10 Administrative Data. Medical Care 2005;43:1130-1139.^e^ Elixhauser A, Steiner C, Kruzikas. D. HCUP Methods Series Report # 2004–1. ONLINE February 6, 2004. U.S. Agency for Healthcare Research and Quality. [Internet]. Comorbidity Software Documentation. Rockville, MD, USA; 2004 [cited 2013]. p. 12–5. Available from: http://www.hcup-us.ahrq.gov/reports/ComorbiditySoftwareDocumentationFinal.pdf. The top 5 most frequent Elixhauser comorbidities identified in the TRD cohort were reported. ^f^ American Psychiatric Association. Diagnostic and statistical manual of mental disorders: DSM-V. Amer Psychiatric Pub Inc; 2013. The top 5 most frequent mental disorders identified in the TRD cohort were reported. ^g^ Depression diagnoses included the following diagnoses ICD-9-CM: 296.2x (MDD—single episode), 296.3x (MDD—recurrent episode), 300.4x (dysthymic disorder), 309.0x (adjustment disorder with depressed mood), 309.1x (prolonged depressive reaction), and 311.x (depressive disorder, not elsewhere classified) or ICD-10-CM: F32x (MDD—single episode), F33x (MDD—recurrent episode), F341 (dysthymic disorder) and F4321 (adjustment disorder with depressed mood).
(DOCX)

S2 Table Behavioral health-related and depression-related HRU per-patient-per-year during the follow-up period (main analysis). **Abbreviations:** CCI = Charlson comorbidity index; CI = confidence interval; ED = emergency department; HRU = healthcare resource utilization; IRR = incidence rate ratio; MDD = major depressive disorder; TRD = treatment-resistant depression. **Notes:** *: significant at the 5% level. ^a^ IRRs, 95% CIs, and p-values were estimated using a generalized linear model with a negative binomial or a Poisson distribution based on the results of the over dispersion test. Over dispersion was detected for nearly all categories of healthcare resource utilization, resulting in the use of a negative binomial distribution instead of the Poisson distribution. ^b^ An IRR > 1 indicates that the TRD cohort had higher healthcare resource utilization than non-MDD cohort. ^c^ Behavioral health-related HRU were identified using the following ICD-9 CM diagnosis codes: 290.xx– 319.xx and their ICD-10 CM equivalents. ^d^ Depression-related HRU were identified using the following ICD-9 CM diagnosis codes: 296.2x, 296.3x, 300.4x, 309.0x, 309.1x, 311.xx and their ICD-10 CM equivalents.
(DOCX)

S3 Table All-cause, behavioral health-related, and depression-related HRU during the follow-up period in the subset of patients aged ≥65. **Abbreviations:** CCI = Charlson comorbidity index; CI = confidence interval; ED = emergency department; HRU = healthcare resource utilization; IRR = incidence rate ratio; MDD = major depressive disorder; TRD = treatment-resistant depression. **Notes:** *: significant at the 5% level. ^a^ IRRs, 95% CIs, and p-values were estimated using a generalized linear model with a negative binomial or a Poisson distribution based on the results of the over dispersion test. Over dispersion was detected for nearly all categories of healthcare resource utilization, resulting in the use of a negative binomial distribution instead of the Poisson distribution. ^b^ An IRR > 1 indicates that the TRD cohort had higher healthcare resource utilization than non-MDD cohort. ^c^ Behavioral health-related HRU were identified using the following ICD-9 CM diagnosis codes: 290.xx– 319.xx and their ICD-10 CM equivalents. ^d^ Depression-related HRU were identified using the following ICD-9 CM diagnosis codes: 296.2x, 296.3x, 300.4x, 309.0x, 309.1x, 311.xx and their ICD-10 CM equivalents.
(DOCX)

S4 Table All-cause, behavioral health-related, and depression-related healthcare costs per-patient-per-year during the follow-up period for the subset of patients aged ≥65. **Abbreviations:** CI = confidence interval; ED = emergency department; SD = standard deviation. **Notes:** *: significant at the 5% level. ^a^ Unadjusted cost differences were estimated using an ordinary least squares regression model and 95% CIs and p-values were estimated using a non-parametric bootstrap procedure (N = 499). ^b^ A cost difference > 0 indicates that the TRD cohort had higher costs than non-TRD MDD cohort. ^c^ Behavioral health-related medical costs were defined as all costs during a visit with any of the following ICD-9 CM diagnostic codes: 290.xx– 319.xx and their ICD-10 CM equivalent. ^d^ Psychiatric pharmacy costs include the following classes of agents: antidepressants, anxiolytics, anticonvulsants/mood stabilizers, antipsychotics, and other mood stabilizers (e.g., lithium). ^e^ Depression-related medical costs were identified using the following ICD-9 CM diagnosis codes: 296.2x, 296.3x, 300.4x, 309.0x, 309.1x, 311.xx and their ICD-10 CM equivalents.
(DOCX)
