# Correction: Burden of treatment-resistant depression in Medicare: A retrospective claims database analysis

**DOI:** 10.1371/journal.pone.0249731

**Published:** 2021-04-01

**Authors:** Dominic Pilon, Kruti Joshi, John J. Sheehan, Miriam L. Zichlin, Peter Zuckerman, Patrick Lefebvre, Paul E. Greenberg

In the Results subsection of the Abstract. there are numbers reported which are inconsistent with those of the main text. Please see the complete, correct Results subsection of the Abstract here:

Of 29,540 patients with MDD, 3,224 (10.9%) met the study definition of TRD; 157,590 were included in the non-MDD cohort. Matched patients with TRD and non-TRD MDD were, on average, 58.9 and 59.0 years old, respectively. The TRD cohort had higher per-patient-per-year (PPPY) HRU than the non-TRD MDD (e.g., inpatient visits: incidence rate ratio [IRR] = 1.34) and non-MDD cohorts (e.g., inpatient visits: IRR = 1.89, all P<0.001). The TRD cohort had significantly higher total PPPY healthcare costs than the non-TRD MDD cohort ($25,059 vs. $19,945, adjusted cost difference = $3,377) and non-MDD cohort ($25,059 vs. $14,410, adjusted cost difference = $3,675, all P<0.001). Similar results were found for the subset of patients ≥65.

There is an error in Table 1. The values in line 9 “Other/Unknown” are missing. Please see the complete, correct Table 1 here.

**Table 1:** Baseline characteristics of matched^a^ cohorts (main analysis).

	TRD cohort	Non-TRD MDD cohort	Std. diff.^b^ (%)	Non-MDD control cohort	Std. diff.^b^ (%)
	N = 3,224	N = 3,224		N = 3,224	
**Age at index date (years), mean ± SD [median]**	58.9 ± 14.6 [60]	59.0 ± 14.6 [61]	0.9	59.0 ± 14.6 [61]	0.9
**Female, n (%)**	2,064 (64.0)	2,052 (63.6)	0.8	2,066 (64.1)	0.1
**Race, n (%)**					
White	2,645 (82.0)	2,671 (82.8)	2.1	2,654 (82.3)	0.7
Black	328 (10.2)	326 (10.1)	0.2	328 (10.2)	—
Asian	35 (1.1)	25 (0.8)	3.2	28 (0.9)	2.2
Other/Unknown	216 (6.7)	202 (6.3)	1.8	214 (6.6)	0.2
**Year of index date, n (%)**^**c**^					
2011	458 (14.2)	457 (14.2)	0.1	457 (14.2)	0.1
2012	681 (21.1)	679 (21.1)	0.2	678 (21.0)	0.2
2013	497 (15.4)	490 (15.2)	0.6	497 (15.4)	—
2014	475 (14.7)	476 (14.8)	0.1	473 (14.7)	0.2
2015	516 (16.0)	522 (16.2)	0.5	521 (16.2)	0.4
	470 (14.6)	475 (14.7)	0.4	470 (14.6)	—
2017	127 (3.9)	125 (3.9)	0.3	128 (4.0)	0.2
**Geographical region, n (%)**^**d**^					
Northeast	543 (16.8)	540 (16.7)	0.2	545 (16.9)	0.2
Midwest	809 (25.1)	809 (25.1)	—	806 (25.0)	0.2
South	1,308 (40.6)	1,317 (40.8)	0.6	1,312 (40.7)	0.3
West	558 (17.3)	555 (17.2)	0.2	555 (17.2)	0.2
Unknown	<11 (<0.3)	<11 (<0.3)	—	<11 (<0.3)	—
**Quan-CCI, mean ± SD [median]**^**e**^	1.4 ± 1.6 [1]	1.3 ± 1.5 [1]	6.6	1.0 ± 1.3 [1]	28.9
**Top 5 most frequent physical comorbidities, n (%)**^**f**^					
Hypertension	1,955 (60.6)	1,902 (59.0)	3.4	1,559 (48.4)	24.9
Diabetes	924 (28.7)	937 (29.1)	0.9	795 (24.7)	9.1
Chronic pulmonary disease	909 (28.2)	799 (24.8)	7.7	558 (17.3)	26.2
Deficiency anemias	640 (19.9)	575 (17.8)	5.2	404 (12.5)	20.0
Hypothyroidism	564 (17.5)	537 (16.7)	2.2	425 (13.2)	12.0
**Top 5 most frequent mental comorbidities, n (%)**^**g**^					
Depression^h^	1,808 (56.1)	1,947 (60.4)	8.8	187 (5.8)	129.6
Anxiety disorders	1,016 (31.5)	879 (27.3)	9.3	245 (7.6)	63.2
Sleep-wake disorders	764 (23.7)	658 (20.4)	7.9	336 (10.4)	35.9
Substance-related and addictive disorders	702 (21.8)	613 (19.0)	6.9	313 (9.7)	33.6
Other conditions that may be a focus of clinical attention	500 (15.5)	446 (13.8)	4.7	235 (7.3)	26.1
**Baseline costs and resource use**					
**Had ≥1 healthcare visit/service, n (%)**					
Inpatient	825 (25.6)	705 (21.9)	8.8	324 (10.0)	41.5
ED	1,109 (34.4)	961 (29.8)	9.8	592 (18.4)	37.0
Outpatient	3,030 (94.0)	3,050 (94.6)	2.7	2,848 (88.3)	20.0
Other	1,729 (53.6)	1,578 (48.9)	9.4	1,454 (45.1)	17.1
**Total healthcare costs (US $2017), mean ± SD [median]**	26,498 ± 57,243 [7,236]	22,064 ± 54,182 [5,215]	8.0	11,564 ± 27,935 [3,017]	33.2
Medical costs	23,745 ± 56,246 [5,098]	19,403 ± 52,085 [3,380]	8.0	9,094 ± 25,439 [1,749]	33.6
Pharmacy costs	2,753 ± 8,779 [950]	2,661 ± 9,751 [697]	1.0	2,470 ± 9,568 [543]	3.1

**Abbreviations:** ED = emergency department; MDD = major depressive disorder; Quan-CCI = Quan-Charlson comorbidity index; SD = standard deviation; Std. diff. = standardized difference; TRD = treatment-resistant depression

Notes

^a^Patients were matched on propensity score (the probability of being in the TRD cohort vs. the non-TRD MDD or non-MDD cohort), generated using probability estimates from a logistic regression model adjusted for categorical age, sex, race, year of the index date, geographical region, and type of healthcare plan

^b^For continuous variables, the standardized difference is calculated by dividing the absolute difference in means of the control and the TRD cohorts by the pooled standard deviation of both groups. The pooled standard deviation is the square root of the average of the squared standard deviations. For dichotomous variables, the standardized difference is calculated using the following equation where P is the respective proportion of participants in each group: (PTRD-Pcontrol)/√[(PTRD(1-PTRD)+Pcontrol(1-Pcontrol))/2].

^c^The index date was defined as the date of the first prescription fill for an antidepressant.

^d^Based on U.S. census regions (http://www2.census.gov/geo/pdfs/maps-data/maps/reference/us_regdiv.pdf) [27].

^e^Quan H, Sundararajan V, Halfon P et al. Coding Algorithms for Defining Comorbidities in ICD-9-CM and ICD-10 Administrative Data. Medical Care 2005;43:1130–1139 [28].

^f^Elixhauser A, Steiner C, Kruzikas. D. HCUP Methods Series Report # 2004–1. ONLINE February 6, 2004. U.S. Agency for Healthcare Research and Quality. [Internet]. Comorbidity Software Documentation. Rockville, MD, USA; 2004 [cited 2013]. p. 12–5. Available from: http://www.hcup-us.ahrq.gov/reports/ComorbiditySoftwareDocumentationFinal.pdf [29]. The top 5 most frequent Elixhauser comorbidities identified in the TRD cohort were reported.

^g^American Psychiatric Association. Diagnostic and statistical manual of mental disorders: DSM-V. Amer Psychiatric Pub Inc; 2013 [29]. The top 5 most frequent mental disorders identified in the TRD cohort were reported [30].

^h^Depression diagnoses included the following diagnoses ICD-9-CM: 296.2x (MDD—single episode), 296.3x (MDD—recurrent episode), 300.4x (dysthymic disorder), 309.0x (adjustment disorder with depressed mood), 309.1x (prolonged depressive reaction), and 311.x (depressive disorder, not elsewhere classified) or ICD-10-CM: F32x (MDD—single episode), F33x (MDD—recurrent episode), F341 (dysthymic disorder) and F4321 (adjustment disorder with depressed mood).

In the Baseline demographic and clinical characteristics subsection of the Results section, there are errors in the sixth sentence of the first paragraph. The correct sentence is:

The mean duration of the observation period was 21.6, 20.7, and 19.1 months in the TRD, non-TRD MDD, and the non-MDD cohort, respectively.

In the Costs subsection of the Results section, there is an error in the first sentence of the second paragraph. The correct sentence is:

All-cause PPPY medical costs drove the majority of the cost difference whether TRD patients were compared to those with non-TRD MDD or non-MDD (% of total adjusted cost difference: vs. TRD = 75.7%, vs. non-TRD MDD = 73.5%); outpatient costs were the main driver for both comparisons (Table 2).

In the Discussion section, there is an error in the second sentence of the fourth paragraph. The correct sentence is:

However, the difference appeared smaller than that observed in different populations (Medicare = $3,377, commercial = $6,709, Medicaid = $4,382) [12, 18], suggesting the incremental cost burden of TRD may be lower in Medicare-insured patients.
