# Supplementary figures and images for: Burden of treatment-resistant depression in Medicare: A retrospective claims database analysis
Source: PLoS One. 2019 Oct 10;14(10):e0223255. doi: 10.1371/journal.pone.0223255 (PMC6786597; doi:10.1371/journal.pone.0223255)

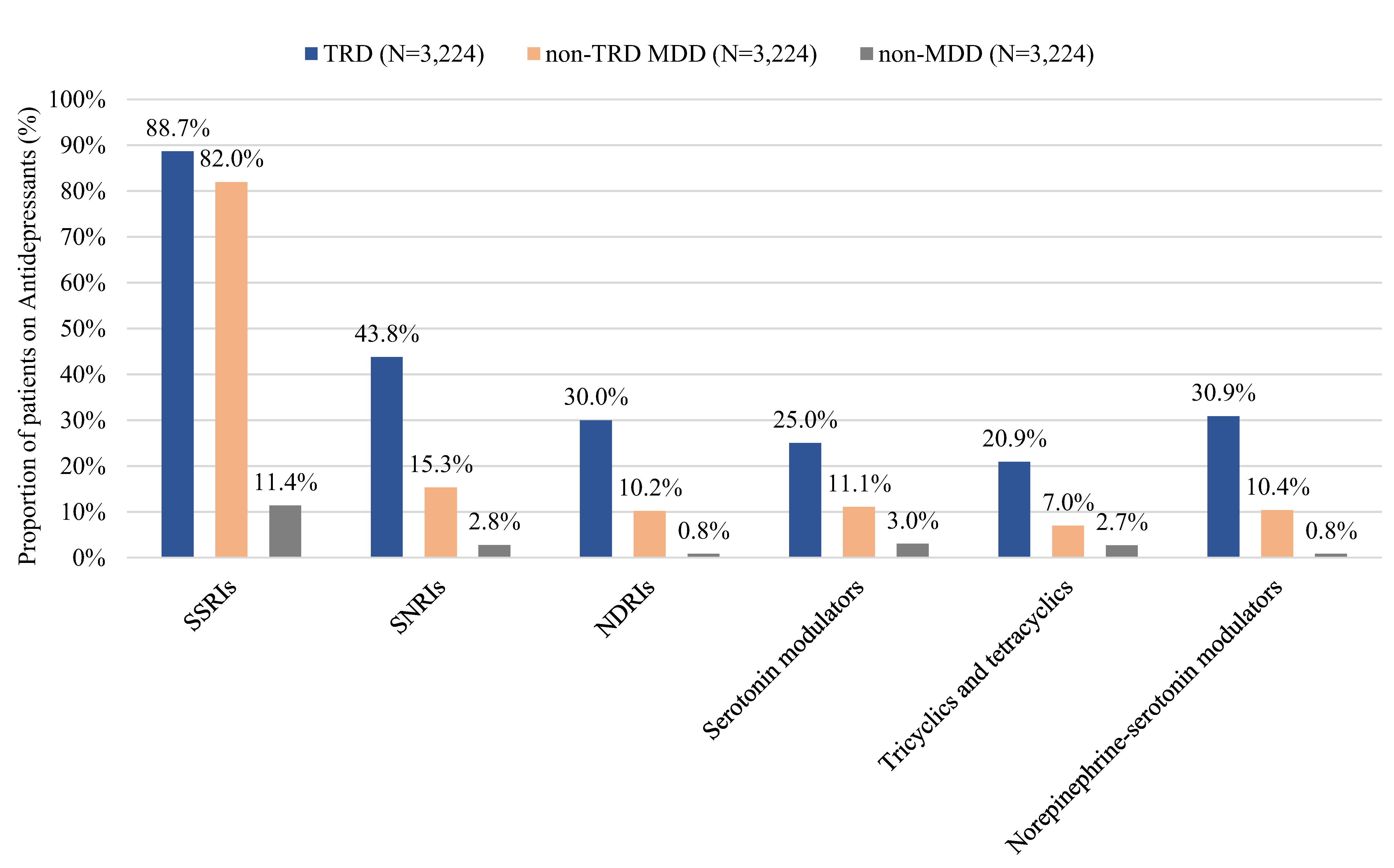

Supplement: S1 Fig — Abbreviations: MDD = major depressive disorder; NDRI = norepinephrine-dopamine reuptake inhibitors; SSRI = selective serotonin reuptake inhibitors; SNRI = serotonin-norepinephrine reuptake inhibitors; TRD = treatment-resistant depression. (TIF) [file pone.0223255.s001.tif]
