# Supplementary material for: Burden of treatment-resistant depression in Medicare: A retrospective claims database analysis
Source: PLoS One. 2019 Oct 10;14(10):e0223255. doi: 10.1371/journal.pone.0223255 (PMC6786597; doi:10.1371/journal.pone.0223255)
Supplement: S1 Table — Abbreviations: ED = emergency department; MDD = major depressive disorder; Quan-CCI = Quan-Charlson comorbidity index; SD = standard deviation; Std. diff. = standardized difference; TRD = treatment-resistant depression. Notes: a For continuous variables, the standardized difference is calculated by dividing the absolute difference in means of the control and the TRD cohorts by the pooled standard deviation of both groups. The pooled standard deviation is the square root of the average of the squared standard deviations. For dichotomous variables, the standardized difference is calculated using the following equation where P is the respective proportion of participants in each group: (PTRD-Pcontrol)/√[(PTRD(1-PTRD)+Pcontrol(1-Pcontrol))/2]. b The index date was defined as the date of the first prescription fill for an antidepressant. c Based on U.S. census regions (http://www2.census.gov/geo/pdfs/maps-data/maps/reference/us_regdiv.pdf). d Quan H, Sundararajan V, Halfon P et al. Coding Algorithms for Defining Comorbidities in ICD-9-CM and ICD-10 Administrative Data. Medical Care 2005;43:1130-1139.e Elixhauser A, Steiner C, Kruzikas. D. HCUP Methods Series Report # 2004–1. ONLINE February 6, 2004. U.S. Agency for Healthcare Research and Quality. [Internet]. Comorbidity Software Documentation. Rockville, MD, USA; 2004 [cited 2013]. p. 12–5. Available from: http://www.hcup-us.ahrq.gov/reports/ComorbiditySoftwareDocumentationFinal.pdf. The top 5 most frequent Elixhauser comorbidities identified in the TRD cohort were reported. f American Psychiatric Association. Diagnostic and statistical manual of mental disorders: DSM-V. Amer Psychiatric Pub Inc; 2013. The top 5 most frequent mental disorders identified in the TRD cohort were reported. g Depression diagnoses included the following diagnoses ICD-9-CM: 296.2x (MDD—single episode), 296.3x (MDD—recurrent episode), 300.4x (dysthymic disorder), 309.0x (adjustment disorder with depressed mood), 309.1x (prolonged depressive rea [file pone.0223255.s002.docx]

**S1 Table.** Patient baseline characteristics (main analysis) before matching

|  | **TRD cohort** | **Non-TRD MDD cohort** | **Std. diff.^1^  (%)** | **Non-MDD control cohort** | **Std. diff.^1^  (%)** | | |
| --- | --- | --- | --- | --- | --- | --- | --- |
|  | **(N=3,224)** | **(N=26,316)** |  | **(N=157,590)** |  |  |  |
| **Age at index date (years), mean ± SD [median]** | 58.9 ± 14.6 [60] | 66.0 ± 13.9 [68] | 49.6 | 70.6 ± 11.7 [71] | | 88.3 |  |
| **Female, n (%)** | 2,064 (64.0) | 17,097 (65.0) | 2.0 | 86,839 (55.1) | | 18.2 |  |
| **Race, n (%)** |  |  |  |  | |  |  |
| White | 2,645 (82.0) | 22,142 (84.1) | 5.6 | 127,994 (81.2) | | 2.1 |  |
| Black | 328 (10.2) | 2,293 (8.7) | 5.0 | 16,528 (10.5) | | 1.0 |  |
| Asian | 35 (1.1) | 345 (1.3) | 2.1 | 3,875 (2.5) | | 10.4 |  |
| Other/Unknown | 216 (6.7) | 1,536 (5.8) | 3.6 | 9,193 (5.8) | | 3.6 |  |
| **Year of index date, n (%)^2^** |  |  |  |  | |  |  |
| 2011 | 458 (14.2) | 3,090 (11.7) | 7.3 | 11,437 (7.3) | | 22.6 |  |
| 2012 | 681 (21.1) | 4,551 (17.3) | 9.7 | 20,176 (12.8) | | 22.3 |  |
| 2013 | 497 (15.4) | 3,556 (13.5) | 5.4 | 20,275 (12.9) | | 7.3 |  |
| 2014 | 475 (14.7) | 3,644 (13.8) | 2.5 | 23,145 (14.7) | | 0.1 |  |
| 2015 | 516 (16.0) | 4,235 (16.1) | 0.2 | 27,258 (17.3) | | 3.5 |  |
| 2016 | 470 (14.6) | 4,799 (18.2) | 9.9 | 33,869 (21.5) | | 18.1 |  |
| 2017 | 127 (3.9) | 2,441 (9.3) | 21.6 | 21,430 (13.6) | | 34.7 |  |
| **Geographical region, n (%)^3^** |  |  |  |  | |  |  |
| Northeast | 543 (16.8) | 4,701 (17.9) | 2.7 | 30,095 (19.1) | | 5.9 |  |
| Midwest | 809 (25.1) | 6,424 (24.4) | 1.6 | 37,079 (23.5) | | 3.6 |  |
| South | 1,308 (40.6) | 10,549 (40.1) | 1.0 | 61,118 (38.8) | | 3.7 |  |
| West | 558 (17.3) | 4,603 (17.5) | 0.5 | 28,905 (18.3) | | 2.7 |  |
| Unknown | <11 (<0.3) | 39 (0.1) | -- | 293 (0.2) | | -- |  |
| **Quan-CCI, mean ± SD [median]^4^** | 1.4 ± 1.6 [1] | 1.5 ± 1.6 [1] | 2.6 | 1.1 ± 1.3 [1] | | 25.9 |  |
| **Top 5 most frequent physical comorbidities, n (%)^5^** |  |  |  |  | |  |  |
| Hypertension | 1,955 (60.6) | 17,552 (66.7) | 12.6 | 91,754 (58.2) | | 4.9 |  |
| Diabetes | 924 (28.7) | 7,959 (30.2) | 3.5 | 41,482 (26.3) | | 5.2 |  |
| Chronic pulmonary disease | 909 (28.2) | 6,590 (25.0) | 7.1 | 23,942 (15.2) | | 31.9 |  |
| Deficiency anemias | 640 (19.9) | 5,236 (19.9) | 0.1 | 20,930 (13.3) | | 17.7 |  |
| Hypothyroidism | 564 (17.5) | 5,130 (19.5) | 5.2 | 23,370 (14.8) | | 7.2 |  |
| **Top 5 most frequent mental comorbidities, n (%)^6^** |  |  |  |  | |  |  |
| Depression^7^ | 1,808 (56.1) | 15,492 (58.9) | 5.6 | 5,435 (3.4) | | 140.8 |  |
| Anxiety disorders | 1,016 (31.5) | 6,925 (26.3) | 11.5 | 8,131 (5.2) | | 72.4 |  |
| Sleep-wake disorders | 764 (23.7) | 5,358 (20.4) | 8.1 | 13,838 (8.8) | | 41.3 |  |
| Substance-related and addictive disorders | 702 (21.8) | 3,713 (14.1) | 20.1 | 7,947 (5.0) | | 50.7 |  |
| Other conditions that may be a focus of clinical attention | 500 (15.5) | 3,435 (13.1) | 7.0 | 10,094 (6.4) | | 29.5 |  |
| **Baseline costs and resource use** |  |  |  |  | |  |  |
| **Had ≥1 healthcare visit/service, n (%)** |  |  |  |  | |  |  |
| Inpatient | 825 (25.6) | 6,231 (23.7) | 4.4 | 14,872 (9.4) | | 43.5 |  |
| ED | 1,109 (34.4) | 7,221 (27.4) | 15.1 | 20,802 (13.2) | | 51.4 |  |
| Outpatient | 3,030 (94.0) | 25,134 (95.5) | 6.8 | 143,139 (90.8) | | 11.9 |  |
| Other | 1,729 (53.6) | 14,423 (54.8) | 2.4 | 74,117 (47.0) | | 13.2 |  |
| **Total healthcare costs (US $2017), mean ± SD [median]** | 26,498 ± 57,243 [7,236] | 24,993 ± 57,960 [5,909] | 2.6 | 9,967 ± 24,760 [2,935] | | 37.5 |  |
| Medical costs | 23,745 ± 56,246 [5,098] | 22,563 ± 56,063 [4,176] | 2.1 | 8,223 ± 22,964 [1,747] | | 36.1 |  |
| Pharmacy costs | 2,753 ± 8,779 [950] | 2,431 ± 13,282 [760] | 2.9 | 1,744 ± 7,342 [428] | | 12.5 |  |

**Abbreviations:** ED = emergency department; MDD = major depressive disorder; Quan-CCI = Quan-Charlson comorbidity index; SD = standard deviation; Std. diff. = standardized difference; TRD = treatment-resistant depression

**Notes:**

[1] For continuous variables, the standardized difference is calculated by dividing the absolute difference in means of the control and the TRD cohorts by the pooled standard deviation of both groups. The pooled standard deviation is the square root of the average of the squared standard deviations. For dichotomous variables, the standardized difference is calculated using the following equation where P is the respective proportion of participants in each group: (PTRD-Pcontrol)/√[(PTRD(1-PTRD)+Pcontrol(1-Pcontrol))/2].

[2] The index date was defined as the date of the first prescription fill for an antidepressant.

[3] Based on U.S. census regions (<http://www2.census.gov/geo/pdfs/maps-data/maps/reference/us_regdiv.pdf>).

[4] Quan H, Sundararajan V, Halfon P et al. Coding Algorithms for Defining Comorbidities in ICD-9-CM and ICD-10 Administrative Data. Medical Care 2005;43:1130-1139.

[5] Elixhauser A, Steiner C, Kruzikas. D. HCUP Methods Series Report # 2004-1. ONLINE February 6, 2004. U.S. Agency for Healthcare Research and Quality. [Internet]. Comorbidity Software Documentation. Rockville, MD, USA; 2004 [cited 2013]. p. 12–5. Available from: <http://www.hcup-us.ahrq.gov/reports/ComorbiditySoftwareDocumentationFinal.pdf>. The top 5 most frequent Elixhauser comorbidities identified in the TRD cohort were reported.

[6] American Psychiatric Association. Diagnostic and statistical manual of mental disorders: DSM-V. Amer Psychiatric Pub Inc; 2013. The top 5 most frequent mental disorders identified in the TRD cohort were reported.

[7] Depression diagnoses included the following diagnoses ICD-9-CM: 296.2x (MDD - single episode), 296.3x (MDD - recurrent episode), 300.4x (dysthymic disorder), 309.0x (adjustment disorder with depressed mood), 309.1x (prolonged depressive reaction), and 311.x (depressive disorder, not elsewhere classified) or ICD-10-CM: F32x (MDD - single episode), F33x (MDD - recurrent episode), F341 (dysthymic disorder) and F4321 (adjustment disorder with depressed mood).
