# Supplementary material for: Burden of treatment-resistant depression in Medicare: A retrospective claims database analysis
Source: PLoS One. 2019 Oct 10;14(10):e0223255. doi: 10.1371/journal.pone.0223255 (PMC6786597; doi:10.1371/journal.pone.0223255)
Supplement: S2 Table — Abbreviations: CCI = Charlson comorbidity index; CI = confidence interval; ED = emergency department; HRU = healthcare resource utilization; IRR = incidence rate ratio; MDD = major depressive disorder; TRD = treatment-resistant depression. Notes: *: significant at the 5% level. a IRRs, 95% CIs, and p-values were estimated using a generalized linear model with a negative binomial or a Poisson distribution based on the results of the over dispersion test. Over dispersion was detected for nearly all categories of healthcare resource utilization, resulting in the use of a negative binomial distribution instead of the Poisson distribution. b An IRR > 1 indicates that the TRD cohort had higher healthcare resource utilization than non-MDD cohort. c Behavioral health-related HRU were identified using the following ICD-9 CM diagnosis codes: 290.xx– 319.xx and their ICD-10 CM equivalents. d Depression-related HRU were identified using the following ICD-9 CM diagnosis codes: 296.2x, 296.3x, 300.4x, 309.0x, 309.1x, 311.xx and their ICD-10 CM equivalents. (DOCX) [file pone.0223255.s003.docx]

**S2 Table.** Behavioral health-related and depression-related HRU per-patient-per-year during the follow-up period (main analysis)

| **HRU per patient per year** | **TRD cohort**  **(N=3,224)** | **Non-TRD MDD cohort**  **(N=3,224)** | **IRR adjusted for baseline costs and CCI (95% CI)^1,2^** | ***P*-value** | **Non-MDD cohort**  **(N=3,224)** | **IRR adjusted for baseline costs and CCI (95% CI)^1,2^** | ***P*-value** |
| --- | --- | --- | --- | --- | --- | --- | --- |
| **Behavioral health-related^3^** |  |  |  |  |  |  |  |
| Inpatient visits | 0.49 | 0.33 | 1.49 (1.36 - 1.64) | <0.001* | 0.11 | 3.84 (3.39 - 4.34) | <0.001* |
| Number of days | 2.77 | 1.82 | 1.57 (1.37 - 1.80) | <0.001* | 0.59 | 3.93 (3.34 - 4.63) | <0.001* |
| ED visits | 0.50 | 0.38 | 1.35 (1.22 - 1.50) | <0.001* | 0.13 | 3.77 (3.33 - 4.27) | <0.001* |
| Outpatient visits | 5.66 | 3.49 | 1.63 (1.53 - 1.73) | <0.001* | 0.57 | 9.70 (8.95 - 10.52) | <0.001* |
| Other visits | 1.16 | 0.70 | 1.63 (1.36 - 1.95) | <0.001* | 0.13 | 6.42 (5.19 - 7.94) | <0.001* |
| **Depression-related^4^** |  |  |  |  |  |  |  |
| Inpatient visits | 0.34 | 0.22 | 1.53 (1.37 - 1.71) | <0.001* | 0.03 | 10.88 (8.95 - 13.22) | <0.001* |
| Number of days | 2.00 | 1.23 | 1.68 (1.43 - 1.98) | <0.001* | 0.15 | 14.04 (11.49 - 17.15) | <0.001* |
| ED visits | 0.23 | 0.17 | 1.38 (1.21 - 1.58) | <0.001* | 0.02 | 12.87 (10.10 - 16.39) | <0.001* |
| Outpatient visits | 3.90 | 2.31 | 1.69 (1.57 - 1.82) | <0.001* | 0.12 | 30.88 (27.65 - 34.49) | <0.001* |
| Other visits | 0.91 | 0.53 | 1.66 (1.36 - 2.04) | <0.001* | 0.06 | 13.11 (10.15 - 16.93) | <0.001* |

**Abbreviations:** CCI = Charlson comorbidity index; CI = confidence interval; ED = emergency department; HRU = healthcare resource utilization; IRR = incidence rate ratio; MDD = major depressive disorder; TRD = treatment-resistant depression

**Notes:**

*: significant at the 5% level

[1] IRRs, 95% CIs, and p-values were estimated using a generalized linear model with a negative binomial or a Poisson distribution based on the results of the over dispersion test. Over dispersion was detected for nearly all categories of healthcare resource utilization, resulting in the use of a negative binomial distribution instead of the Poisson distribution.

[2] An IRR > 1 indicates that the TRD cohort had higher healthcare resource utilization than non-MDD cohort.

[3] Behavioral health-related HRU were identified using the following ICD-9 CM diagnosis codes: 290.xx – 319.xx and their ICD-10 CM equivalents.

[4] Depression-related HRU were identified using the following ICD-9 CM diagnosis codes: 296.2x, 296.3x, 300.4x, 309.0x, 309.1x, 311.xx and their ICD-10 CM equivalents.
