# Supplementary material for: Burden of treatment-resistant depression in Medicare: A retrospective claims database analysis
Source: PLoS One. 2019 Oct 10;14(10):e0223255. doi: 10.1371/journal.pone.0223255 (PMC6786597; doi:10.1371/journal.pone.0223255)
Supplement: S3 Table — Abbreviations: CCI = Charlson comorbidity index; CI = confidence interval; ED = emergency department; HRU = healthcare resource utilization; IRR = incidence rate ratio; MDD = major depressive disorder; TRD = treatment-resistant depression. Notes: *: significant at the 5% level. a IRRs, 95% CIs, and p-values were estimated using a generalized linear model with a negative binomial or a Poisson distribution based on the results of the over dispersion test. Over dispersion was detected for nearly all categories of healthcare resource utilization, resulting in the use of a negative binomial distribution instead of the Poisson distribution. b An IRR > 1 indicates that the TRD cohort had higher healthcare resource utilization than non-MDD cohort. c Behavioral health-related HRU were identified using the following ICD-9 CM diagnosis codes: 290.xx– 319.xx and their ICD-10 CM equivalents. d Depression-related HRU were identified using the following ICD-9 CM diagnosis codes: 296.2x, 296.3x, 300.4x, 309.0x, 309.1x, 311.xx and their ICD-10 CM equivalents. (DOCX) [file pone.0223255.s004.docx]

**S3 Table.** All-cause, behavioral health-related, and depression-related HRU during the follow-up period in the subset of patients aged ≥65

| **HRU per patient per year** | **TRD cohort**  **(N=1,338)** | **Non-TRD MDD cohort**  **(N=1,338)** | **IRR adjusted for baseline costs and CCI (95% CI)^1,2^** | ***P*-value** | **Non-MDD cohort**  **(N=1,338)** | **IRR adjusted for baseline costs and CCI (95% CI)^1,2^** | ***P*-value** |
| --- | --- | --- | --- | --- | --- | --- | --- |
| **Behavioral health-related^3^** |  |  |  |  |  |  |  |
| Inpatient visits | 0.49 | 0.31 | 1.54 (1.33 - 1.77) | <0.001* | 0.08 | 4.94 (4.05 - 6.02) | <0.001* |
| Number of days | 2.88 | 1.68 | 1.67 (1.36 - 2.05) | <0.001* | 0.47 | 4.34 (3.36 - 5.59) | <0.001* |
| ED visits | 0.31 | 0.18 | 1.66 (1.39 - 1.99) | <0.001* | 0.04 | 6.08 (4.71 - 7.86) | <0.001* |
| Outpatient visits | 5.46 | 2.86 | 1.90 (1.71 - 2.12) | <0.001* | 0.28 | 17.85 (15.47 - 20.59) | <0.001* |
| Other visits | 1.66 | 0.78 | 1.87 (1.47 - 2.38) | <0.001* | 0.08 | 14.29 (10.60 - 19.27) | <0.001* |
| **Depression-related^4^** |  |  |  |  |  |  |  |
| Inpatient visits | 0.37 | 0.23 | 1.60 (1.36 - 1.87) | <0.001* | 0.02 | 12.30 (9.08 - 16.67) | <0.001* |
| Number of days | 2.14 | 1.23 | 1.74 (1.38 - 2.20) | <0.001* | 0.14 | 13.34 (9.85 - 18.07) | <0.001* |
| ED visits | 0.18 | 0.11 | 1.68 (1.36 - 2.08) | <0.001* | 0.01 | 11.64 (7.73 - 17.53) | <0.001* |
| Outpatient visits | 3.81 | 2.06 | 1.86 (1.65 - 2.10) | <0.001* | 0.07 | 48.18 (39.54 - 58.70) | <0.001* |
| Other visits | 1.30 | 0.62 | 1.86 (1.42 - 2.42) | <0.001* | 0.04 | 24.31 (16.94 - 34.88) | <0.001* |

**Abbreviations:** CCI = Charlson comorbidity index; CI = confidence interval; ED = emergency department; HRU = healthcare resource utilization; IRR = incidence rate ratio; MDD = major depressive disorder; TRD = treatment-resistant depression

**Notes:**

*: significant at the 5% level

[1] IRRs, 95% CIs, and p-values were estimated using a generalized linear model with a negative binomial or a Poisson distribution based on the results of the over dispersion test. Over dispersion was detected for nearly all categories of healthcare resource utilization, resulting in the use of a negative binomial distribution instead of the Poisson distribution.

[2] An IRR > 1 indicates that the TRD cohort had higher healthcare resource utilization than non-MDD cohort.

[3] Behavioral health-related HRU were identified using the following ICD-9 CM diagnosis codes: 290.xx – 319.xx and their ICD-10 CM equivalents.

[4] Depression-related HRU were identified using the following ICD-9 CM diagnosis codes: 296.2x, 296.3x, 300.4x, 309.0x, 309.1x, 311.xx and their ICD-10 CM equivalents.
