# Supplementary material for: Burden of treatment-resistant depression in Medicare: A retrospective claims database analysis
Source: PLoS One. 2019 Oct 10;14(10):e0223255. doi: 10.1371/journal.pone.0223255 (PMC6786597; doi:10.1371/journal.pone.0223255)
Supplement: S4 Table — Abbreviations: CI = confidence interval; ED = emergency department; SD = standard deviation. Notes: *: significant at the 5% level. a Unadjusted cost differences were estimated using an ordinary least squares regression model and 95% CIs and p-values were estimated using a non-parametric bootstrap procedure (N = 499). b A cost difference > 0 indicates that the TRD cohort had higher costs than non-TRD MDD cohort. c Behavioral health-related medical costs were defined as all costs during a visit with any of the following ICD-9 CM diagnostic codes: 290.xx– 319.xx and their ICD-10 CM equivalent. d Psychiatric pharmacy costs include the following classes of agents: antidepressants, anxiolytics, anticonvulsants/mood stabilizers, antipsychotics, and other mood stabilizers (e.g., lithium). e Depression-related medical costs were identified using the following ICD-9 CM diagnosis codes: 296.2x, 296.3x, 300.4x, 309.0x, 309.1x, 311.xx and their ICD-10 CM equivalents. (DOCX) [file pone.0223255.s005.docx]

**S4 Table.** All-cause, behavioral health-related, and depression-related healthcare costs per-patient-per-year during the follow-up period for the subset of patients aged ≥65

| **Healthcare cost (US $2017) per patient per year** | **Mean ± SD [median]** | | |  | **Adjusted cost difference (95% CI); P-value^1^** | |
| --- | --- | --- | --- | --- | --- | --- |
|  | **TRD cohort**  **(N=1,338)** | **Non-TRD MDD cohort**  **(N=1,338)** | **Non-MDD cohort**  **(N=1,338)** |  | **Non-TRD MDD cohort** | **Non-MDD cohort** |
| **All-cause medical and pharmacy costs** | 26,885 ± 35,671 [13,875] | 19,831 ± 33,446 [8,121] | 12,418 ± 25,778 [4,160] |  | 4524 (2,363 ; 6,918); <0.001* | 7126 (5,024 ; 9,097); <0.001* |
| **All-cause medical costs** | 23,513 ± 33,004 [11,064] | 17,439 ± 31,803 [6,434] | 10,697 ± 24,166 [2,988] |  | 3816 (1,862 ; 5,856); <0.001* | 6375 (4,469 ; 8,273); <0.001* |
| Inpatient costs | 10,091 ± 20,713 [1,127] | 7,597 ± 21,294 [0] | 4,586 ± 16,392 [0] |  | 1442 (-71 ; 2,870); 0.064 | 2552 (1,210 ; 3,829); <0.001* |
| ED costs | 957 ± 2,304 [246] | 661 ± 1,636 [0] | 355 ± 1,281 [0] |  | 221 (76 ; 365); <0.001* | 409 (276 ; 550); <0.001* |
| Outpatient costs | 8,037 ± 12,059 [4,667] | 5,873 ± 9,876 [3,184] | 3,798 ± 7,795 [1,894] |  | 1531 (873 ; 2,270); <0.001* | 2452 (1,689 ; 3,067); <0.001* |
| Other costs | 4,429 ± 9,061 [854] | 3,308 ± 8,523 [389] | 1,958 ± 7,984 [117] |  | 622 (6 ; 1,244); 0.044* | 962 (274 ; 1,644); 0.016* |
| **All-cause pharmacy costs** | 3,371 ± 8,087 [1,854] | 2,392 ± 8,805 [1,168] | 1,721 ± 6,446 [634] |  | 708 (6 ; 1,368); 0.048* | 751 (163 ; 1,368); 0.012* |
| **Behavioral health-related medical and pharmacy costs** | 8,039 ± 13,761 [2,323] | 5,192 ± 12,464 [567] | 1,541 ± 9,679 [0] |  | 2301 (1,334 ; 3,284); <0.001* | 5134 (4,212 ; 6,139); <0.001* |
| **Behavioral health-related medical costs^2^** | 7,576 ± 13,730 [1,805] | 5,027 ± 12,448 [369] | 1,508 ± 9,670 [0] |  | 2006 (1,063 ; 2,954); <0.001* | 4710 (3,804 ; 5,730); <0.001* |
| Inpatient costs | 4,742 ± 10,142 [0] | 3,188 ± 9,711 [0] | 1,222 ± 8,046 [0] |  | 1203 (391 ; 1,912); <0.001* | 2587 (1,891 ; 3,302); <0.001* |
| ED costs | 351 ± 1,675 [0] | 191 ± 752 [0] | 52 ± 446 [0] |  | 143 (53 ; 241); <0.001* | 243 (165 ; 327); <0.001* |
| Outpatient costs | 1,002 ± 2,489 [304] | 542 ± 1,733 [96] | 85 ± 681 [0] |  | 422 (261 ; 582); <0.001* | 838 (726 ; 961); <0.001* |
| Other costs | 1,480 ± 4,548 [0] | 1,106 ± 4,402 [0] | 149 ± 2,495 [0] |  | 237 (-85 ; 563); 0.172 | 1042 (771 ; 1,272); <0.001* |
| **Psychiatric pharmacy costs^2^** | 464 ± 823 [193] | 165 ± 485 [40] | 33 ± 174 [0] |  | 295 (249 ; 348); <0.001* | 424 (380 ; 470); <0.001* |
| **Depression-related pharmacy and medical costs^3^** | 5,729 ± 11,103 [1,138] | 3,801 ± 10,501 [291] | 512 ± 6,216 [0] |  | 1539 (762 ; 2,285); <0.001* | 4289 (3,546 ; 4,995); <0.001* |
| **Depression-related medical costs^3^** | 5,476 ± 11,105 [707] | 3,687 ± 10,487 [137] | 493 ± 6,207 [0] |  | 1401 (623 ; 2,137); <0.001* | 4059 (3,312 ; 4,778); <0.001* |
| Inpatient visits | 3,398 ± 8,591 [0] | 2,295 ± 8,361 [0] | 370 ± 4,772 [0] |  | 854 (211 ; 1,429); 0.008* | 2374 (1,818 ; 2,928); <0.001* |
| ED visits | 214 ± 1,015 [0] | 124 ± 640 [0] | 14 ± 214 [0] |  | 78 (15 ; 143); 0.016* | 168 (116 ; 226); <0.001* |
| Outpatient costs | 710 ± 2,047 [165] | 402 ± 1,597 [44] | 31 ± 515 [0] |  | 278 (120 ; 430); <0.001* | 632 (543 ; 743); <0.001* |
| Other costs | 1,154 ± 3,649 [0] | 866 ± 3,805 [0] | 78 ± 1,756 [0] |  | 191 (-84 ; 465); 0.160 | 884 (667 ; 1,090); <0.001* |
| **Antidepressant pharmacy costs** | 253 ± 369 [109] | 114 ± 266 [29] | 19 ± 113 [0] |  | 137 (112 ; 161); <0.001* | 231 (208 ; 251); <0.001* |

**Abbreviations:** CI = confidence interval; ED = emergency department; SD = standard deviation

**Notes:**

*: significant at the 5% level

[1] Unadjusted cost differences were estimated using an ordinary least squares regression model and 95% CIs and p-values were estimated using a non-parametric bootstrap procedure (N=499).

[2] A cost difference > 0 indicates that the TRD cohort had higher costs than non-TRD MDD cohort.

[3] Behavioral health-related medical costs were defined as all costs during a visit with any of the following ICD-9 CM diagnostic codes: 290.xx – 319.xx and their ICD-10 CM equivalent.
